# LEAPdb: a database for the late embryogenesis abundant proteins

**DOI:** 10.1186/1471-2164-11-221

**Published:** 2010-04-01

**Authors:** Gilles Hunault, Emmanuel Jaspard

**Affiliations:** 1 Université d'Angers, Laboratoire d'Hémodynamique, Interaction Fibrose et Invasivité tumorale hépatique, UPRES 3859, IFR 132, Université d'Angers, F-49045 Angers, France; 2 Université d'Angers, UMR 1191 Physiologie Moléculaire des Semences, IFR 149 QUASAV, F-49045 Angers, France

## Abstract

**Background:**

Late Embryogenesis Abundant Proteins database (LEAPdb) contains resource regarding LEAP from plants and other organisms. Although LEAP are grouped into several families, there is no general consensus on their definition and on their classification. They are associated with abiotic stress tolerance, but their actual function at the molecular level is still enigmatic. The scarcity of 3-D structures for LEAP remains a handicap for their structure-function relationships analysis. Finally, the growing body of published data about LEAP represents a great amount of information that needs to be compiled, organized and classified.

**Results:**

LEAPdb gathers data about 8 LEAP sub-families defined by the PFAM, the Conserved Domain and the InterPro databases. Among its functionalities, LEAPdb provides a browse interface for retrieving information on the whole database. A search interface using various criteria such as sophisticated text expression, amino acids motifs and other useful parameters allows the retrieving of refined subset of entries. LEAPdb also offers sequence similarity search. Information is displayed in re-ordering tables facilitating the analysis of data. LEAP sequences can be downloaded in three formats. Finally, the user can submit his sequence(s). LEAPdb has been conceived as a user-friendly web-based database with multiple functions to search and describe the different LEAP families. It will likely be helpful for computational analyses of their structure - function relationships.

**Conclusions:**

LEAPdb contains 769 non-redundant and curated entries, from 196 organisms. All LEAP sequences are full-length. LEAPdb is publicly available at http://forge.info.univ-angers.fr/~gh/Leadb/index.php.

## Background

"Late Embryogenesis Abundant" proteins (LEAP) were originally discovered in germinating cotton *Gossypium hirsutum *seeds [1-5]. They were also found in the seeds of many other plants as well as various plant tissues. The common unifying trait for the presence of these proteins is their association with abiotic stress tolerance, particularly dehydration, cold stress and salt stress [3,6-8].

Although widely distributed among plants, LEAP have also been found in other organisms. The presence of a dehydration-induced LEA-like gene in a desiccation-tolerant animal such as a nematode [9,10] or an arthropod [11] indeed suggests a general protective role in anhydrobiotic organisms.

LEAP were first classified into five major groups on the basis of their primary sequences [5,7,12]. This classification has been often re-examined using statistically based bioinformatics tools [13,14]. However, no clear criteria for a universal classification of LEAP has emerged (Additional file 1 - Table S1).

LEAP are highly hydrophilic proteins with repeated amino acid motifs and a propensity for alpha-helix formation [15]. Indeed, a LEAP from pea was shown to achieve a high content of amphipathic helices upon dehydration, interacting then with membranes [16]. One possible role of these secondary structures could be to protect membranes during freezing and desiccation [17].

Why are LEAP so intriguing?

(i) They represent a wide family of proteins (found in various organisms as well as in different cellular compartments), itself subdivided in 8 sub-families as defined by the PFAM database [18].

(ii) The structures of LEAP are almost unknown: most of them can be predicted to be natively unfolded, explaining the lack of 3-D structures. One can thus consider that no 3-D structure is currently available within this protein family.

(iii) Very little is known about the molecular mechanism of action of LEAP. Two dehydrins (group 2 LEAP), ERD10 and ERD14, have been shown to be potent molecular chaperones [19]. Studies using mutant LEAP support the hypothesis that the Lys-rich consensus sequences (named the K-segments) of this type of LEAP constitute the interface through which they bind the surface of membranes enriched in anionic phospholipids [20]. However, despite some theoretical studies such as molecular dynamics simulations [10], the actual functional mechanism of LEAP at the molecular level remains to be demonstrated for most of them (*i.e*., no clear partner or cellular target has been yet identified).

Investigating the structure-function relationships of LEAP is thus of primary interest, but remains challenging because experimental evidence is difficult to obtain. Computational analyses of LEAP sequences offer an alternative promising avenue [21] for which a dedicated database would be of primary importance.

The interest toward LEAP is increasing because of their intriguing structural and functional features, leading to the discovery of new types of LEAP, as for example in the case of a new dehydrin pattern from *Tuber borchii *[22] or the two forms (differing mainly by an internal deletion) of a LEAP from a bdelloid rotifer [23]. Concerning the nucleotide sequences, it was demonstrated that more than 50 LEAP-encoding genes in the *Arabidopsis thaliana *genome could be classified into nine distinct groups [24,25].

The growing body of published data about LEAP represents a great amount of information that needs to be compiled, organized and classified. One purpose of LEAPdb is to provide the scientific community a curated archive of LEAP families to navigate, interpret, and understand this enormous amount of data. LEAPdb has been conceived as a user-friendly web-based database with multiple functions to search, describe and analyze LEAP. It will help for the comprehension of the function of this enigmatic family of proteins. It is considered that a lot of LEAP are part of a more widespread family of proteins called hydrophilins whose physiological role is far from being completely understood [26]. A better knowledge of LEAP will lead to that of hydrophilins. In the case of plants and anhydrobiotic species, LEAPdb may contribute to the development of models for unraveling mechanisms used to overcome water loss, freezing or salt-induced stress.

## Construction and content

### Organization of LEAPDB

The user can select any entries through various parameters and conduct further analyses using the implemented tools. For this purpose, LEAPdb has three main features: (i) the browse mode that allows the user to consult all or part of the database; (ii) the search mode based upon multiple search criteria; (iii) the export mode to retrieve sequences in different formats.

### The browse mode

**Figure 1:**
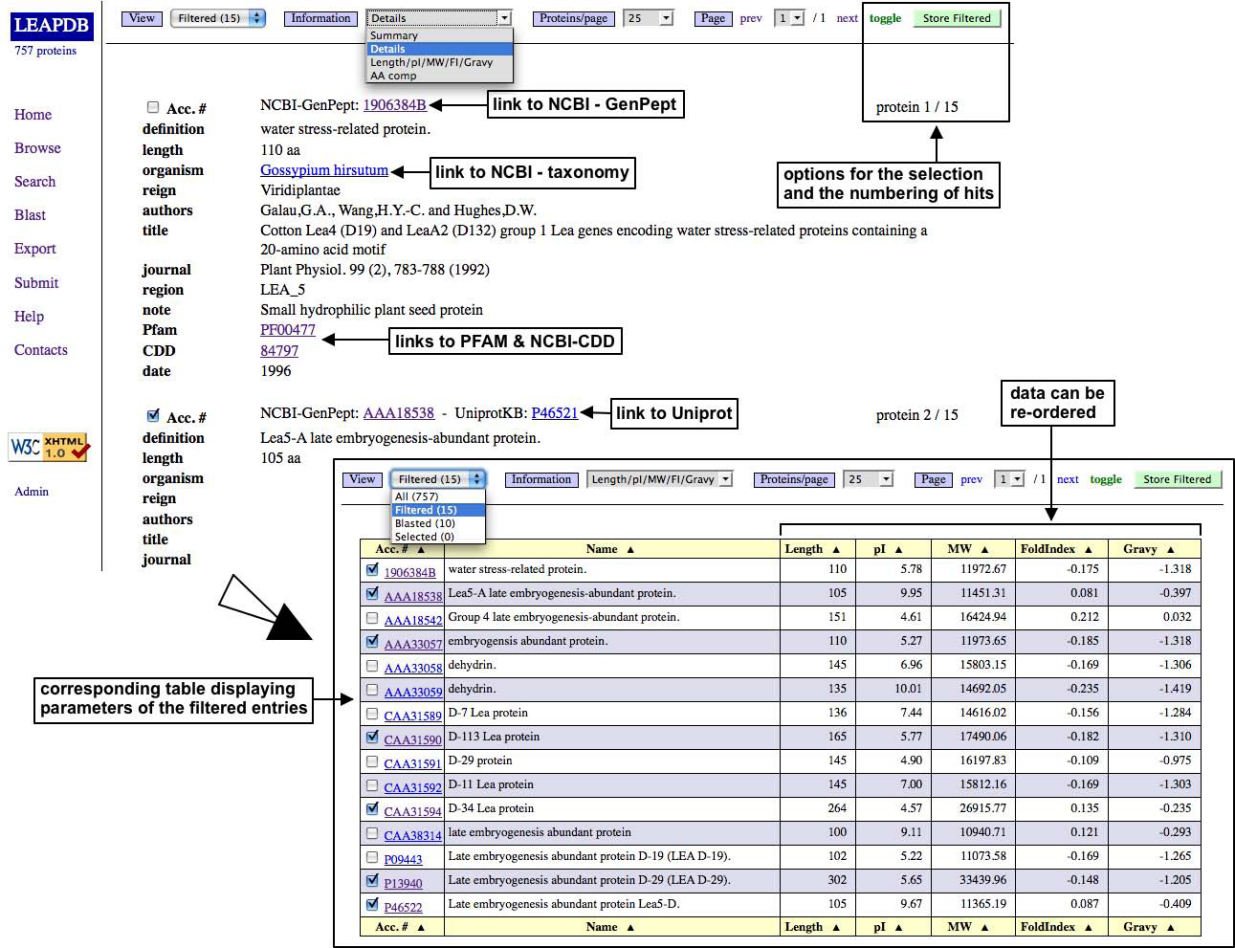
**Browse interface for accessing the whole LEAPdb content**. Example of the detailed annotation of LEAPdb entries. Other types of information can be displayed (Summary, [Length/pI/MW/Fold Index/Gravy] and the amino acids composition). Tables can be re-ordered for a better comparison and analysis of data.

It allows consulting the whole database (Figure 1). The « *Summary *» option provides the NCBI-GenPept accession number and the Uniprot accession number, the name of the sequence and of the organism, the putative function of the LEAP (if any). The « *Details *» option provides more information (up to 20 fields from the GenPept or the Uniprot files). The accession numbers, the name of the organism, the PFAM, the CDD [27] and the InterPro [28] numbers provide a link to their relevant website. A series of physicochemical properties are given by selecting the « *Length/pI/MW/Fold Index/Gravy *» option (the number of amino acids, the isoelectric point, the molecular weight, the fold index [29] and the grand average of hydropathy [30], respectively). Finally, the « *AA comp *» option displays the amino acid composition of LEAP.

### The search mode

**Figure 2:**
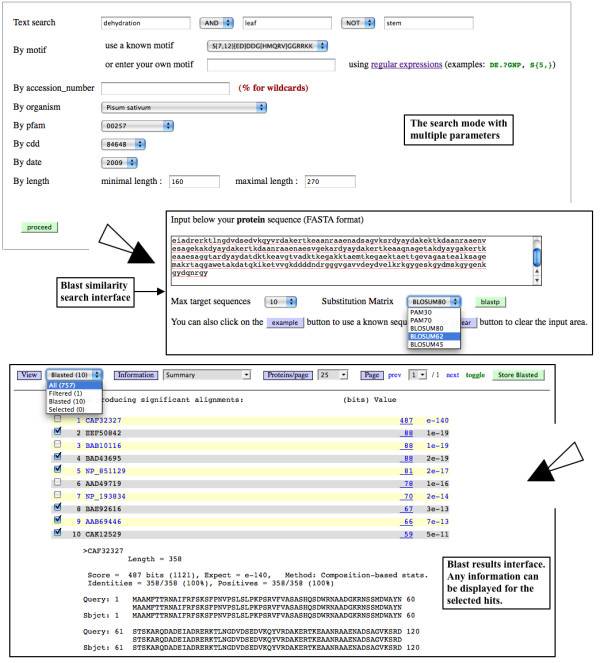
**The search mode**. The search mode is an advanced search with multiple parameters that permits retrieval of very fine subsets of data. Any protein or nucleotide sequence can be analyzed by BLAST similarity search. Same information as those displayed by the browse mode can be viewed for the selected hits of the BLAST result.

It corresponds to an advanced search with multiple parameters and permits retrieval of very fine subsets of data (example of search: "LEAP of the PF00257 family from *Arabidopsis thaliana*, containing the motif *S*{5}, associated to the key word dehydration and found in leaf, published by someone called "Carpenter" in 2003, with a length comprised between 150 and 200 amino acids") (Figure 2). The search can be made as following: by using a sophisticated text expression accepting wildcards and Booleans - this search applies to all fields of the whole database; by accession number (accepting wildcards) - one or multiple accession number(s) separated by a space can be searched at one time; by organism; by PFAM or CDD numbers; by date; by range of length of amino acids sequence. The user can also retrieve sequences by entering amino acids motif either exact or degenerated using regular expressions with sophisticated syntax, allowing to retrieve fine motifs. The known motifs implemented in LEAPdb result from our expertise (Additional file 1 - Table S2).

The user can perform a similarity search by using BLASTP [31] implemented in LEAPdb (Figure 2). This allows obtaining additional information and retrieving the best scoring sequences through the classical BLAST output interface. It is also possible to BLASTX a nucleotide sequence against LEAPdb to check the existence of similar or homologous LEAP in order, for example, to construct a putative LEAP cDNA sequence from EST [32].

Many options are proposed for the output: (i) when entries have been retrieved, the information can be displayed in any of the views of the browse mode and the fields such as accession number, taxonomy, PFAM, CDD and InterPro numbers are linked to their original web site according to the relevant information found at the NCBI; (ii) the user can select or deselect any entry resulting from his search ("*toggle*" option) and the final selection can be stored. This is true for all displays (browse mode, BLAST output, ...); (iii) one original point is the re-ordering (ascending or descending sort) of data displayed in tables, for a better comparison and analysis of data.

### The export mode

All sequences in LEAPdb can be exported trough the "*Export*" interface. The "*View*" menu displays the entries in the order of the last actions made by the user (all, filtered, selected or blasted). The "*Format*" menu displays three exporting formats: Fasta, XML and Excel (CSV).

### The submission form

The user can submit his sequence(s) trough a submission form. After verification of their relevance, they will be integrated in LEAPdb.

### Construction and characteristics of the dataset

The main sources to fill LEAPdb are information contained in "*GenPept*" files and in the corresponding Uniprot files. The primary request to get "*GenPept*" files from the public database NCBI in order to fill LEAPdb contained 471 keywords but was so complex that it overwhelmed the possibility of treatment by the online form and the "*Preview*" option of NCBI interface. A program was written to take into account the most efficient words and restrains the request to groups of 40 words (as suggested by the "*Entrez*" - NCBI developers) and to use the "*History*" option of NCBI interface. LEAPdb contains also some proteins induced by several stress (cold, water, salt and abcisic acid) and some glycine rich proteins.

This allowed the retrieving of more than 4,000 "*GenPept*" files, but with a high redundancy of sequences for many organisms (for example, LEAPdb contains 362 entries for *Arabidopsis thaliana *corresponding to only 71 non-redundant LEAP for this organism - Additional file 1 - Table S3) as well as wrongly LEAP-annotated files.

The pertinence of the files annotation was manually verified and using BLAST homology analysis. Sequence conflict (in particular in the case of AGI entries for *Arabidopsis thaliana*) was checked using annotations from the Uniprot files. Redundant sequences were removed after multiple alignments using Multalin [33] and ClustalW [34] with various parameters (matrix and gap penalties). Hundred of alignments on different crossed pools of sequences selected by different criteria were performed. This allowed detecting truncated or "false" LEAP sequences that were removed from LEAPDB (most of them being furthermore annotated "putative LEA", "unnamed protein product" ...). Seven sequences containing undefined amino acid (X) were also removed.

To fill LEAPdb, we used a two-stage process. The first step filled automatically the fields of the tables of the database using PHP and perl scripts, getting the information from text and xml files from the following databases: NCBI/Proteins, NCBI/CDD, NCBI/Taxonomy, EBI/Picr, UNIPROT, Interpro, SANGER/Pfam, AMIGO. Starting with the NCBI accession number, we got the "*GenPept*" file to derive the GI number, the eventual PFAM, CDD and Interpro identifiers and the textual information. Then, using the cross-references of EBI/Picr we obtained the Uniprot accession number and name to complete and double-check the data. The second step was a thorough manual check to ensure that all information and links were pertinent and relevant to the LEAP issue.

Finally, LEAPdb contains 769 curated, non-redundant and full-length LEAP sequences, from 196 organisms (Additional file 1 - Table S4).

LEAPdb contains also roughly 1790 non-accessible entries, classified as "not wanted" either because they are redundant LEAP files or because they are wrongly annotated. This dataset is very important when the database is updated and for further analyses of LEAP since it can be considered as a "negative control".

## Utility and Discussion

### Benefits of LEAPdb

To our knowledge, there is no database for LEAP and since LEAPdb offers a unique set of curated data of these proteins, it is therefore an invaluable tool to compile, organize and classify the steadily growing body of information concerning LEAP. Its multiple functionalities and tools are making LEAPdb a useful resource for the *in silico *exploration of the structure-function relationship of LEAP on the basis of their primary sequence as well as their various physicochemical properties. Examples of such analysis are given in Additional file 1 - Tables 5 and 6. For this purpose, the link to PFAM, CDD and InterPro is important to provide information about the putative structure of LEAP. The user can also retrieve sequences containing conserved or degenerated amino acids motifs with the « *Search by motif *» option (either by using known regular expressions - Additional file 1 - Table S2, or by entering personal motifs). With the [Blastp/Blastx] interface, it provides a powerful tool for the detection of structural LEAP features.

LEAPdb would also contribute to a better classification of LEAP. For example, 83 LEAP entries contain the annotation "seed maturation protein". However, 51 entries are annotated PF03760 and 7 entries are annotated CDD112567 (PF03760 and CDD112567 correspond to the "LEA_1" family). Among these 83 LEAP entries, only 20 really belong to PF04927 (called "seed maturation protein" in PFAM database). This underlines the ambiguity of old classifications (Additional file 1 - Table S1). It appears that, today, the best classification of LEAP is the one based on conjugated parameters such as the PFAM, CDD and InterPro numbers. This is a step towards a greater integration of the knowledge of the 2-D and the 3-D structure of these proteins that are not yet available. Presently, 81%, 52% and 78% of entries in LEAPdb are PFAM-, CDD- and InterPro-annotated, respectively, and we are making efforts to increase this annotation. LEAPdb is a collection that can help to refine LEAP classification by investigating their taxonomic distribution (especially for plants) or by studying the evolutionary history of the different LEAP sub-families.

A classification tool for LEAP is still missing. Thus we are working on it since each class of LEAP sub-families can be characterized by an unique set of multiple physico-chemical values contained in LEAPdb, making it possible to precisely classify LEAP. It will also help to re-annotate files poorly annotated (e.g., containing keywords like putative protein, uncharacterized protein or unnamed protein).

After examining the 108 sequences of the PFAM PF00477 dataset http://pfam.sanger.ac.uk//family/pf00477, it appears that 12 entries are redundant. Thus it would correspond to 96 non-redundant sequences only. This redundancy is true for some other LEAP-PFAM families, for instance PF00257 that contains 876 sequences among which more than 100 sequences are redundant. This underlines the good curation of LEAPdb.

Why does LEAPdb contain, for example, 58 PF00477 sequences instead of 108? (i) Some entries were not PFAM-annotated at the moment the files were uploaded in LEAPdb; (ii) our expertise makes us consider some entries as non-true LEAP (*i.e.*, some PFAM entries do not actually belong to the LEAP families); (iii) some PFAM entries are fragments of sequences. It is also true in Uniprot [35]: there are more than 1960 hits for the keyword « LEA » in UniprotKB among which there are dozens of fragments (e.g., the 452 sequence clusters - UniRef). Some of them are part of full-length sequences also stored with different accession numbers thus increasing the redundancy of data and decreasing the pertinence/precision of the results for further analysis (such as the search of amino acids signature or statistical calculation). Since we have made the choice to keep only full-length amino acid sequences, LEAPdb does not contain fragments. Therefore, LEAPdb provides complementary information to the big generalist databases.

### Example of use of LEAPdb: the re-analysis of LEAP from Arabidopsis thaliana provides evidence that two LEAP 3D structures are available

**Figure 3:**
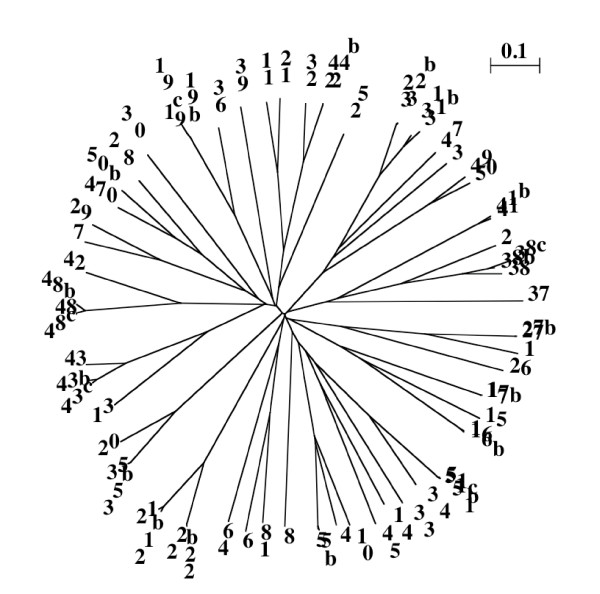
**Re-analysis of LEAP from Arabidopsis thaliana**. Unrooted dendogram of the 71 *Arabidopsis thaliana *LEAP. Sequences were aligned using ClustalW (Gonnet matrix).

We have found 71 LEAP belonging to the different PFAM families (Additional file 1 - Table S3). For a better comparison, the numbering of LEAP in this file is rigorously the same as in Table 1 of the article of Hundertdmark and Hincha about the LEAP-encoding genes in the *Arabidopsis thaliana *genome [25]. The sequences were aligned using ClustalW [34] and a dendrogram was drawn (Figure 3).

Despite the general idea that the structures of LEAP are almost unknown, two LEAP 3D structures are available: PDB # 1XO8 - [36,37] - coded by AT1G01470 and PDB # 1YYC coded by AT2G46140. Both were isolated from *Arabidopsis thaliana *and both are member of the PFAM family PF03168 (LEA_2).

Unfortunately, these two proteins have been wrongly "un-classified" as LEAP for years [14].

The work of Hundertmark and Hincha [25] and our present work clearly demonstrate that AT1G01470 coding for protein #NP_171654 is a true LEAP, and it is easy to verify that this protein is 1XO8. Moreover, the analysis of 1XO8 shows that it is very similar to LEAP #NP_182137 and #AAA18542. It must be mentioned that there are 8 accession numbers for this protein: 1XO8A, AAF81307, AAL75906, AAT71983, CAA71174, CAA73311, O03983. The 8 entries have been stored in LEAPdb (to prevent from any false addition when the database is updated) but only one is accessible (O03983 - Additional file 1 - Table S3).

**Figure 4:**
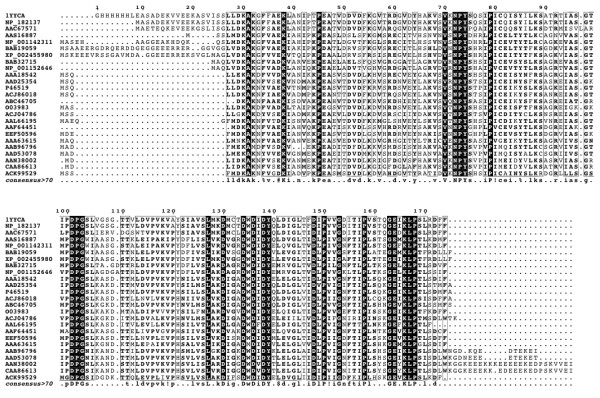
**The "WHy" domain of LEAP**. Alignment of 25 LEAP from the PFAM family PF03168 containing the « WHy » domain. Among them, the two crystallized LEAP O03983 - 1XO8 and 1YYC. The alignment was made using Multalin and the figure was drawn using ESPript 2.2.

A novel domain has been found in the LEAP family PF03168, which is named « WHy » for « Water stress and Hypersensitive response domain » [38]. Interestingly, two members of this family are 1XO8 and 1YYC (Figure 4). It is therefore possible to modelize putative 3D-structure of LEAP family PF03168 using the X-ray data in order to answer some questions such as: is there a common molecular mechanism of response to external stresses for different proteins families? Is there a similar pathway for the stress response for LEAP from plants, bacteria and archaea?

### Future developments and perspectives

For quantitative data (*i.e.*, length of sequences/pI/MW/Fold Index/Gravy/frequency of amino acids and many other physico-chemical properties), classical statistical computation and graphs will be automatically generated.

We are currently developing software for the analysis of sequence submitted by the users in order to predict if it belongs to any of the LEAP families and to provide them a complete set of information. This software will also be an efficient classification tool for LEAP.

The main goal of LEAPdb is the analysis of the structure-function relationships of LEAP. This explains why amino acids sequences were first implemented. However, genomic data (genes and pseudogenes), coding sequences (cDNA or mRNA) and expression data (EST) will be soon incorporated, in order to help exploring some crucial issues: how many LEAP are encoded in each particular organism? Which of these proteins are encoded by separate genes? Which result from alternative splicing? Nevertheless, today the user can BLASTX any nucleotide sequence against LEAPdb to check the existence of similar or homologous LEAP.

Since deciphering the molecular functions of LEAP is a major issue, we will provide lexical tools (dictionaries by alphabetic order or occurrence or synonyms...) for a better semantic analysis of the words that describe the known elements of the function of LEAP.

## Conclusions

LEAPdb harbors a comprehensive data set available for late embryogenesis abundant proteins, together with tools designed for their online analysis. To our knowledge, there is no equivalent database for LEAP. LEAPdb will thus constitute an efficient tool (i) for the compilation and the organization of growing data concerning LEAP and, more generally, hydrophilins; (ii) for the classification of the various sub-families of LEAP; (iii) for the design of experiments to elucidate the function of this enigmatic proteins; (iv) to help the analysis of the LEAP structure-function relationships.

## Availability and requirements

Project name: LEAPdb: a database for the late embryogenesis abundant proteins.

Project home page: http://forge.info.univ-angers.fr/~gh/Leadb/index.php.

Operating system(s): Platform independent.

Programming language: LEAPdb is a fast and interactive Web-based database with a user-friendly interface written in PHP. Some options need javascript to be activated. Information is provided to the user from a MySQL relational database. The statistical computations are performed using the R software called by a PHP script.

Use by non-academics: no licence needed.

## Authors' contributions

GH implemented the MySQL database, PHP and Perl scripts, and the interface design. EJ collected data, checked data integrity and entered them into LEAPdb. EJ served as project advisor. GH and EJ wrote the manuscript, checked the accuracy of the database and web interface, read and approved the final manuscript.

## Supplementary Material

Additional file 1 **Tables of the article**. Table S1: Main classifications of LEAP with time. Evolution of the classification of LEAP initially started by Dure and his colleague who discovered them. Now, the best classification is the PFAM numbering. **Table S2: Structural characteristics of LEAP**. PFAM, CDD and Interpro numbers and specific motif sequence of each LEAP family. The amount of LEAP found in LEAPdb for each motif is compared to the one found by scanning UniProtKB/Swiss-Prot, UniProtKB/TrEMBL. **Table S3: The 71 LEAP entries from *Arabidopsis thaliana *in LEAPdb**. For a better comparison, the numbering of LEAP is rigorously the same as in Table 1 of the article of Hundertdmark and Hincha [25]. **Table S4: Taxonomy of the organisms in LEAPdb**. The amount of LEAP is indicated within the 196 organisms in LEAPdb. **Table S5: Some physico-chemical properties of LEAP**. The minimum and the maximum values of the amino acids sequence length, the molecular weight (MW), the isoelectric point (pI), the Fold Index (FI) and the grand average of hydropathy (Gravy) is indicated for each specific motif sequence found in the different LEAP families. **Table S6: Main characteristics of the amino acids composition of LEAP**. The range of percentage of some specific amino acids is calculated over the total number of LEAP in LEAPdb retrieved using the indicated motif. Click here for file
