# Supplementary material for: LEAPdb: a database for the late embryogenesis abundant proteins
Source: BMC Genomics. 2010 Apr 1;11:221. doi: 10.1186/1471-2164-11-221 (PMC2858754; doi:10.1186/1471-2164-11-221)
Supplement: Additional file 1 — Tables of the article. Table S1: Main classifications of LEAP with time. Evolution of the classification of LEAP initially started by Dure and his colleague who discovered them. Now, the best classification is the PFAM numbering. Table S2: Structural characteristics of LEAP. PFAM, CDD and Interpro numbers and specific motif sequence of each LEAP family. The amount of LEAP found in LEAPdb for each motif is compared to the one found by scanning UniProtKB/Swiss-Prot, UniProtKB/TrEMBL. Table S3: The 71 LEAP entries from Arabidopsis thaliana in LEAPdb. For a better comparison, the numbering of LEAP is rigorously the same as in Table 1 of the article of Hundertdmark and Hincha [25]. Table S4: Taxonomy of the organisms in LEAPdb. The amount of LEAP is indicated within the 196 organisms in LEAPdb. Table S5: Some physico-chemical properties of LEAP. The minimum and the maximum values of the amino acids sequence length, the molecular weight (MW), the isoelectric point (pI), the Fold Index (FI) and the grand average of hydropathy (Gravy) is indicated for each specific motif sequence found in the different LEAP families. Table S6: Main characteristics of the amino acids composition of LEAP. The range of percentage of some specific amino acids is calculated over the total number of LEAP in LEAPdb retrieved using the indicated motif. [file 1471-2164-11-221-S1.DOC]

**Table 1.** Main classifications of LEAP with time.

| PFAM  (name) | Dure et al.^a^ | Bray | Tunnacliffe and Wise | Battaglia et al. | Bies-Esthève et al. | Hundertmark and Hincha | This work | Some LEAP names |
| --- | --- | --- | --- | --- | --- | --- | --- | --- |
| PF00257  (dehydrin) | D11 | Group 2 | Group 2 | Group 2 | Group 2 | dehydrin | PF00257 | Cor47 / ERD10 / ERD14 / LTI29 / LTI30 / Xero 1 / Xero 2 / RAB18 |
| PF04927  (SMP^b^) | D34 | Group 6 | Group 6 | Group 5A | Group 5 | SMP | PF04927 | RAB28 / MtPM25 |
| PF03760  (LEA_1) |  | Group 4 | Group 4 | Group 4A | Group 4 | LEA_1 | PF03760 | PAP260 / PAP051 |
|  | D113 |  |  | Group 4B |  |  |  |  |
| PF03168  (LEA_2) | D95 | --- | --- | Group 5C | Group 7 | LEA_2 | PF03168 | Lea14 (1XO8) / 1YYC |
| PF03242  (LEA_3) | D73 | --- | Lea5 | Group 5B | Group 6 | LEA_3 | PF03242 | AtD121 |
| PF02987  (LEA_4) | D7 | Group 3 | Group 3 | Group 3A | Group 6 | LEA_4 | PF02987 | COR15 / PvLEA1 / AfrLEA / Aavlea1 |
|  | D29 | Group 5 |  | Group 3B |  |  |  |  |
| PF00477  (LEA_5) | D19  D132 | Group 1 | Group 1 | Group 1 | Group 1 | LEA_5 | PF00477 | EM1 / EM6 |
| PF10714  (LEA_6) | --- | --- | --- | Group 6 | Group 8 | PvLEA18 | PF10714 | PvLEA18 |
| PF10714  (ABA-WDS) | --- | --- | --- | Group 7 | --- | --- | --- | ASR |

^a^ References: Dure et al., 1989 - Bray, 1993 - Tunnacliffe and Wise, 2007 - Battaglia et al., 2008 - Bies-Ethève et al., 2008 - Hundertmark and Hincha, 2008

^b^ Seed maturation proteins.

**Table 2.** Structural characteristics of LEAP.

| LEAP  family | PFAM  CDD  Interpro | Motif N° | Motif sequence^a^ | Number of LEAP retrieved^b^ | ScanProsite result^c^ |
| --- | --- | --- | --- | --- | --- |
| dehydrin | PF00257  CDD84648  CDD109320  IPR000167 | 1 | [DEG][KR]I[KM][DE]K[IL][PR]G | 245 | 303  sequences |
|  |  | 2 | Subset 1: SGS{4,}EDDG | 110 | d |
|  |  | 3 | Subset 2: [AGST][DGNST]S{5,}DE{1,} | 57 | d |
| SMP | PF04927  CDD68499  CDD113691  IPR007011 | 4 | T[ILV][GT][ER]AL[EHK]A | 20 | 127 |
| LEA_1 | PF03760  CDD67378  CDD112567  IPR005513  IPR007011 | 5 | [AS][EKNST][AILMV][DEGQ][EG]K[AGT]E[KR][AM][KMRT][AT][HQR] | 60 | 67 |
|  |  | 6 | Subset 1: AK[AS]GM[DE]KTKA | 30 | 31 |
|  |  | 7 | Subset 2:  E[KLR][AS][MRT]ART[EK]EE | 31 | 38 |
| LEA_2 | PF03168  CDD66818  CDD92778  CDD121280  CDD141399  IPR004864 | 8 | Subset 1: NPY.{4,}P[IV].[ADEQ].{2,}[FY] | 30 | 46 |
|  |  | 9 | Subset 2: [DE].{0,1}L.{1,2}NPN[GP][FV].{3,3}L.{4,4}Y | 22 | 26 |
| LEA_3 | PF03242  CDD66886  CDD112072  IPR004926 | 10 | W[AGLMV][PR]DP[AIKPQRTV][RST]G[DFNTY][GWY] | 62 | 63 |
| LEA_4 | PF02987  CD66647  CDD111833  IPR004238 | 11 | AGE[AT][KRT] | 43 | *Not specific* |
| LEA_5 | PF00477  CDD84797  CDD88917  CDD109530  IPR000389 | 12 | [KR]G[AG][ENQT][AT]R[AKR][DEQ]Q | 58 | 84 |
| LEA_6 | PF10714  CDD119234  IPR018930 | 13 | Subset 1: ED[HY]K[KLMQR][KLNQR][AG]YG | 12 | 15 |
|  |  | 14 | Subset 2: T[DE]APT[ILPV][PS]G | 12 | 17 |

^a^Pattern syntax for motifs: «.» = any amino acid; X{n,} = at least n times X; [XYZ] = X or Y or Z.

^b^Number of sequences retrieved in LEAPdb using the motif indicated.

^c^ScanProsite result on all UniProtKB/Swiss-Prot, UniProtKB/TrEMBL, PDB databases sequences, excluding fragments.

^d^Regular expression not compatible with the pattern syntax authorized by ScanProsite.

## Table 3. The 71 LEAP entries for Arabidopsis thaliana in LEAPdb.

|  | AGI code | LEAPDB # | PFAM |  | AGI code | LEAPDB # | PFAM |
| --- | --- | --- | --- | --- | --- | --- | --- |
| a | At1g01470 | O03983 | PF03168 | a | At3g17520 | NP_188379 | PF02987 |
| a | At1g02820 | ABD57466 | PF03242 | d | At3g19430 | N. W. | ----- |
| a | At1g03120 | NP_171811 | PF04927 | a | At3g22490.1 | AAO22723 | PF04927 |
| d | At1g04560 | N. W. | ----- | b | *At3g22490.2?* | CAA63085 | PF04927 |
| a | At1g20440 | NP_173468 | PF00257 | a | At3g22500.1 | AAK96509 | PF04927 |
| a | At1g20450.1 | NP_850947 | PF00257 | b | *At3g22500.2?* | BAA11016 | PF04927 |
| b | At1g20450.2 | NP_564114 | PF00257 | d | At3g50790 | N. W. | ----- |
| d | At1g22600 | N. W. | ----- | a | At3g50970 | AAB00374 | PF00257 |
| a | At1g32560 | CAA61676 | PF03760 | a | At3g50980 | AAB00375 | PF00257 |
| d | At1g34340 | N. W. | ----- | a | At3g51810.1 | NP_190749 | PF00477 |
| a | At1g52690 | NP_175678 | PF02987 | b | *At3g51810.2?* | AAA32825 | PF00477 |
| a | At1g54410 | NP_175843 | PF00257 | a | At3g53040 | AAL59922 | PF02987 |
| d | At1g54890 | N. W. | ----- | a | At3g53770 | AAY78769 | PF03242 |
| d | At1g61340 | N. W. | ----- | a | At4g02380.1 | NP_567231 | PF03242 |
| d | At1g71730 | N. W. | ----- | b | At4g02380.2 | NP_001078346 | PF03242 |
| c | At1g72100 | N. W. | ----- | b | *At4g02380.3?* | BAH57115 | PF03242 |
| a | At1g76180 | AAK00404 | PF00257 | d | At4g05010 | N. W. | ----- |
| d | At2g03140 | N. W. | ----- | d | At4g13235 | N. W. | ----- |
| a | At2g03740 | ABE65800 | PF02987 | a | At4g13230 | NP_567398 | PfamB 64161 |
| a | At2g03850 | NP_565306 | PF02987 | c | At4g13560 | N. W. | ----- |
| a | At2g18340 | AAD15503 | PF02987 | a | At4g15910.1 | NP_193326 | PF03242 |
| a | At2g21490 | AAD23693 | PF00257 | b | At4g15910.2 | AAO24597 | PF03242 |
| a | At2g23110 | NP_179892 | PF10714 | a | At4g21020 | NP_193834 | PfamB 203514 |
| a | At2g23120.1 | NP_565548 | PF10714 | d | At4g27400 | N. W. | ----- |
| b | At2g23120.2 | Q94K79 | PF10714 | a | At4g36600.1 | NP_195378 | PF02987 |
| a | At2g33690.1 | NP_180925 | PF10714 | b | At4g36600.2 | CAB80326 | PF02987 |
| b | At2g33690.2 | AAS47599 | PF10714 | b | At4g36600.3 | AAT06422 | PF02987 |
| a | At2g35300 | AAC61808 | PF03760 | c | At4g38410 | N. W. | ----- |
| a | At2g36640.1 | AAD20140 | PF02987 | a | At4g39130 | NP_195624 | PF00257 |
| b | At2g36640.2 | BAA11017 | PF02987 | a | At5g06760 | NP_196294 | PF03760 |
| b | At2g36640.3 | BAC42987 | PF02987 | a | At5g27980 | NP_198150 | PF04927 |
| a | At2g40170 | CAA77508 | PF00477 | b | AT5G38760 | NP_198692 | PfamB 8736 |
| a | At2g41260.1 | NP_973656 | No PFAM domain | a | At5g44310.1 | BAD43695 | PfamB 35368 |
| b | At2g41260.2 | NP_181659 | No PFAM domain | b | *At5g44310.2?* | NP_851129 | PfamB 35368 |
| a | At2g41280.1 | NP_181660 | No PFAM domain | b | *At5g44310.3?* | BAB10116 | PfamB 35368 |
| b | At2g41280.2 | BAC42584 | No PFAM domain | a | At5g53260 | NP_200138 | PF04927 |
| a | At2g42530 | NP_181781 | PfamB 15227 & PfamB 23356 | a | At5g53270 | NP_200139 | PF04927 |
| a | At2g42540.1 | NP_181782 | PfamB 15227 & PfamB 23356 | b | At5g53820 | NP_200193 | PfamB 8736 |
| b | At2g42540.2 | NP_850371 | PfamB 15227 & PfamB 23356 | d | At5g54370 | N. W. | ----- |
| a | At2g42560 | NP_181784 | PF02987 | d | At5g60520 | N. W. | ----- |
| a | At2g44060 | NP_181934 | PF03168 | d | At5g60530 | N. W. | ----- |
| a | At2g46140.1 | NP_182137 | PF03168 | a | At5g66400.1 | NP_201441 | PF00257 |
| b | At2g46140.2 | 1YYCA | PF03168 | b | *At5g66400.2?* | NP_001032162 | PF00257 |
| a | At3g02480 | NP_566173 | PfamB 8736 | b | *At5g66400.3?* | AAA32722 | PF00257 |
| a | At3g15670 | NP_188188 | PfamB 12553 & PfamB 6460 |  |  |  |  |

a: Included in the study of Hundertmark & Hincha [25] and stored in LEAPdb.

b: Not included in the study of Hundertmark & Hincha but stored in LEAPdb - it corresponds to variant product (alternative splicing, punctual mutation, …).

c: Included in the study of Hundertmark & Hincha but not wanted (N.W.) in LEAPdb (stored non-accessible entries).

d: Not included or rejected in the study of Hundertmark & Hincha (see the Additional file 1 of their article [25]) and N.W. in LEAPdb.

**Table 4.** Taxonomy of the organisms in LEAPdb.

| Taxonomy | Number of proteins | | | |
| --- | --- | --- | --- | --- |
| Archaea | 7 |  |  |  |
| Bacteria | 41 |  |  |  |
| Eukaryota | 713 |  |  |  |
|  | | | | |
| Details of Eukaryota | | | | |
| Alveolata | 5 |  |  |  |
| Fungi/Metazoagroup (*total 26*) | | | | |
|  | Ascomycota | 6 |  |  |
|  | Bilateria | 20 |  |  |
|  |  | Coelomata 8 |  |  |
|  |  |  | Crustacea | 5 |
|  |  |  | Hexapoda | 3 |
|  |  | Pseudocoelomata 12 |  |  |
|  |  |  | Nematoda | 8 |
|  |  |  | Rotifera | 4 |
| Viridiplantae (*total 682*) | | | | |
|  | Chlorophyta | 4 |  |  |
|  | Streptophyta | (*Total 678*) |  |  |
|  |  | Funariidae | 2 |  |
|  |  | Filicophyta | 1 |  |
|  |  | Ginkgoopsida | 1 |  |
|  |  | Coniferopsida | 64 |  |
|  |  | Magnoliophyta | 610 |  |
|  |  |  | Eudicotyledons | 436 |
|  |  |  | Liliopsida | 174 |

**Table 5.** Some physico-chemical properties of LEAP.

| LEAP  family | Motif N° | LEAP | Length  (number of aa) | MW^a^  (g/mol) | pI | FI | Gravy |
| --- | --- | --- | --- | --- | --- | --- | --- |
| dehydrin | 1 | 225 | 218 LEAP: 93 - 295 | 10156 - 31444 | 4.41 - 10.46 | -0.365 to -0.004 | -1.789 to -0.738 |
|  |  | 20 | 19 LEAP: 326 - 639 | 31257 - 69635 | 5.97 - 9.47 | -0.311 to 0.058 | -1.593 to -0.582 |
|  | 2 | 110 | 101 - 566 | 10154 - 60574 | 5.70 - 10.46 | -0.248 to 0.020 | -1.573 to -0.687 |
|  | 3 | 57 | 140 - 292 | 16663 - 27925 | 4.41 - 10.29 | -0.365 to -0.096 | -1.789 to -0.922 |
| SMP | 4 | 20 | 159 - 278 | 15037 - 31094 | 3.94 - 5.28 | 0.001 to 0.166 | -0.564 to -0.103 |
| LEA_1 | 5 | 60 | 88 - 173 | 9260 - 17609 | 8.69 - 10.19 | -0.171 to -0.019 | -1.223 to -0.795 |
|  | 6 | 30 | 88 - 173 | 9260 - 17609 | 8.80 - 10.19 | -0.162 to -0.019 | -1.181 to -0.795 |
|  | 7 | 31 | 126 - 141 | 13736 - 15074 | 8.69 - 10.57 | -0.171 to -0.090 | -1.223 to -0.932 |
| LEA_2 | 8 | 30 | 95 - 180 | 10958 - 19754 | 4.32 - 9.75 | 0.106 to 0.261 | -0.319 to 0.129 |
|  | 9 | 22 | 256 - 368 | 26892 - 41085 | 4.38 - 10.63 | 0.029 to 0.306 | -0.479 to 0.279 |
| LEA_3 | 10 | 62 | 78 - 144 | 8118 - 16019 | 6.36 - 12.20 | -0.043 to 0.168 | -0.790 to -0.167 |
| LEA_4 | 11 | 43 | 115 - 733 | 12290 - 77051 | 4.48 - 11.08 | -0.326 to 0.070 | -1.631 to -0.554 |
| LEA_5 | 12 | 58 | 83 - 182 | 8961 - 19716 | 4.49 - 9.41 | -0.303 to -0.118 | -1.688 to -0.977 |
| LEA_6 | 13  14 | 12 | 71 - 117 | 7556 - 12165 | 4.22 - 9.38 | -0.207 to -0.048 | -1.355 to -0.909 |

^a^MW : molecular weight; pI: isoelectric point; FI: Fold Index; Gravy: grand average of hydropathy

## Table 6. Main characteristics of the amino acids composition of LEAP.

| LEAP  family | Motif N° | LEAP | %A | %C | %[D+E] | %G | %[K+R] | %[S+T] | %W | %[F+W+Y] |
| --- | --- | --- | --- | --- | --- | --- | --- | --- | --- | --- |
| dehydrin | 1  (≤ 295 aa) | 225 | 0.4 to 13.4 | 166 LEAP: no C  52 LEAP: 0.4 to 5.4 | 3.1 to 34.9 | 3.8 to 34.1 | 3.9 to 29.5 | 0.9 to 28.3 | 210 LEAP: no W  8 LEAP: 0.4 to 1.3 | 0 to 12.6 |
|  | 1  (≥ 326 aa) | 20 | 3.5 to 14.7 | 14 LEAP: no C  5 LEAP: 0.4 to 1.1 | 4.8 to 27.5 | 4.4 to 33.1 | 3.7 to 20.7 | 3.7 to 27.5 | 17 LEAP: no W  2 LEAP: 0.5 | 2.6 to 13.0 |
|  | 2 | 110 | 1.3 to 14.7 | 93 LEAP: no C  15 LEAP: 0.2 to 0.5 | 5.2 to 18.5 | 11.4 to 33.9 | 5.6 to 18.1 | 6.0 to 32.8 | 105 LEAP: no W  3 LEAP: 0.6 to 1.3 | 1.1 to 11.0 |
|  | 3 | 57 | 3.4 to 11.4 | 38 LEAP: no C  15 LEAP: 0.4 to 2.0 | 11.4 to 31.5 | 3.8 to 17.9 | 7.2 to 32.6 | 8.0 to 20.4 | 50 LEAP: no W  3 LEAP: 0.4 to 0.9 | 0 to 7.3 |
| SMP | 4 | 20 | 12.5 to 23.9 | 10 LEAP: no C  10 LEAP: 0.4 to 1.3 | 10.0 to 19.6 | 7.4 to 14.4 | 5.1 to 15.7 | 7.0 to 20.3 | 17 LEAP: no W  3 LEAP: 0.4 to 1.1 | 0 to 4.9 |
| LEA_1 | 5 | 60 | 10.1 to 20.5 | 58 LEAP: no C  2 LEAP: 0.7 to 0.8 | 6.1 to 16.2 | 4.5 to 18.8 | 8.6 to 18.3 | 4.9 to 24.7 | no W | 0.8 to 5.6 |
|  | 6 | 30 | 10.1 to 20.5 | no C | 5.3 to 16.0 | 9.6 to 18.8 | 8.8 to 18.1 | 12.9 to 22.4 | no W | 1.1 to 3.6 |
|  | 7 | 31 | 12.2 to 16.3 | 29 LEAP: no C  2 LEAP: 0.7 to 0.8 | 7.5 to 15.4 | 4.5 to 10.6 | 12.0 to 18.2 | 5.9 to 17.7 | no W | 0.8 to 5.6 |
| LEA_2 | 8 | 30 | 4.4 to 9.9 | 3 LEAP: no C  23 LEAP: 0.6 to 2.0 | 7.9 to 22.4 | 0.0 to 10.0 | 5.9 to 16.4 | 8.0 to 19.5 | 2 LEAP: no W  24 LEAP: 0.6 to 2.0 | 2.9 to 13.6 |
|  | 9 | 22 | 3.1 to 11.3 | 11 LEAP: no C  9 LEAP: 0.3 to 1.4 | 6.2 to 21.9 | 6.8 to 12.5 | 4.9 to 19.2 | 6.5 to 14.8 | 4 LEAP: no W  16 LEAP: 0.6 to 0.9 | 4.1 to 10.0 |
| LEA_3 | 10 | 62 | 5.6 to 28.4 | 39 LEAP: no C  16 LEAP: 0.7 to 2.0 | 5.0 to 17.2 | 2.2 to 12.9 | 7.0 to 25.3 | 4.2 to 25.6 | 0.9 to 2.8 | 1.8 to 13.7 |
| LEA_4 | 11 | 43 | 10.2 to 26.5 | 31 LEAP: no C  12 LEAP: 0.2 to 1.8 | 7.7 to 33.6 | 3.5 to 12.3 | 8.3 to 27.8 | 5.1 to 29.6 | 36 LEAP: no W  7 LEAP: 0.4 to 2.0 | 0.0 to 15.5 |
| LEA_5 | 12 | 58 | 3.3 to 13.6 | 55 LEAP: no C  1 LEAP: 1.1 | 12.5 to 25.6 | 14.7 to 23.1 | 7.8 to 24.2 | 1.6 to 22.2 | no W | 0.0 to 6.6 |
| LEA_6 | 13  14 | 12 | 2.5 to 12.0 | no C | 9.3 to 20.8 | 9.4 to 17.9 | 4.3 to 21.7 | 9.9 to 32.9 | no W | 2.4 to 6.8 |
